# Beyond antibiotics: collateral damage to the gut microbiome during cancer therapy

**DOI:** 10.3389/fmicb.2026.1955949

**Published:** 2026-09-21

**Authors:** Tong Jin, Yuanshi Lv, Shanshan Jiang, Yanhua Jia

**Affiliations:** 1 Department of Thyroid and Head and Neck Surgery II, Jilin Provincial Cancer Hospital, Changchun, China; 2 Department of Thyroid and Head and Neck Surgery I, Jilin Provincial Cancer Hospital, Changchun, China; 3 The Ultrasound Department, Jilin Provincial Cancer Hospital, Changchun, China; 4 Department of Integrated Traditional Chinese and Western Medicine, Jilin Provincial Cancer Hospital, Changchun, China

**Keywords:** cancer chemotherapy, collateral microbiome injury, colonization resistance, ecological recovery, ecological resilience, gut microbiome, oncology exposome, pathobiont domination

## Abstract

Antibiotics are major drivers of microbiome disruption in oncology, but they do not fully explain the ecological changes observed during cytotoxic treatment. The central question of this Mini Review is when chemotherapy-associated microbiome change should be interpreted as transient compositional variation and when it constitutes a loss of ecosystem function with potential clinical consequences. We use collateral microbiome injury as a conceptual framework, rather than a formally established clinical diagnosis, to describe treatment-associated loss of microbial load, function, spatial organization, colonization resistance, or recovery capacity that is not required for antitumor activity. Three questions organize the review: which changes are directly attributable to a defined chemotherapeutic agent; which arise from the broader oncology exposome, including mucosal injury, antibiotics, nutritional disturbance, hospitalization, and supportive medications; and which ecological mechanisms are inferred from non-chemotherapy models. The evidence is therefore classified as direct chemotherapy evidence, oncology-exposome evidence, or general ecological evidence. Agent-specific studies support microbial transformation of irinotecan and fluoropyrimidines, whereas longitudinal leukemia studies more often capture composite treatment episodes. Across these settings, depletion of obligate anaerobe functions, altered nutrient and redox conditions, pathobiont domination, oral-to-gut niche coalescence, and impaired recovery may weaken colonization resistance. However, the strongest persistent-disruption and infection data arise from intensive hematologic treatment and should not be generalized uncritically to conventional solid-tumor chemotherapy. We propose operational criteria, multidimensional recovery metrics, and evidence-stratified translational priorities for moving from descriptive dysbiosis toward testable, microbiome-sparing supportive care.

## 1 Introduction

The intestinal microbiota of patients receiving cancer treatment is commonly described as being damaged by antibiotics. This emphasis is justified because antibacterial prophylaxis and empirical treatment are frequent during neutropenia, and agents with broad anaerobic activity can produce profound ecosystem disruption. Nevertheless, an antibiotic-only explanation is incomplete. A large-scale screen showed that many drugs developed for human targets inhibit at least one gut bacterial strain, and subsequent studies demonstrated extensive microbial drug metabolism, inter-individual variation in metabolic capacity, and bacterial bioaccumulation of therapeutic compounds (Maier et al., 2018; Zimmermann et al., 2019; Javdan et al., 2020; Klünemann et al., 2021). Drug-microbiome associations are also combinatorial and dose-dependent, while bacterial transcriptomic responses include transport, efflux, vitamin metabolism, and stress adaptation (Forslund et al., 2021; Ricaurte et al., 2024). These observations support viewing medications as ecological exposures rather than agents acting exclusively on the host.

This perspective is particularly relevant to cytotoxic chemotherapy. Parent compounds or active metabolites may reach the bowel through incomplete absorption, intestinal or biliary excretion, epithelial shedding, and enterohepatic cycling. Simultaneously, chemotherapy changes the habitat through epithelial injury, mucus loss, inflammation, altered food intake, dysmotility, neutropenia, and supportive medications. The central scientific problem is therefore not simply whether the microbiome changes, but which component of a composite treatment episode caused the change, whether a protective ecological function was lost, and whether the altered state persists long enough to affect mucosal recovery, infection risk, or treatment continuity. A 2025 systematic review of 22 human studies likewise found substantial heterogeneity and limited causal evidence, reinforcing the need to separate prediction of treatment response from treatment-associated ecological injury (Böhm et al., 2025).

The human evidence base is asymmetric. Intensive acute-leukemia and transplantation settings provide dense longitudinal sampling and clinically important infection outcomes, but chemotherapy is embedded within hospitalization, profound neutropenia, nutritional disturbance, and repeated broad-spectrum antibiotic exposure. Solid-tumor studies are often more agent-specific and less dominated by prolonged neutropenia, but cohorts remain smaller and regimens heterogeneous. Findings from these settings are therefore complementary rather than interchangeable. This review explicitly separates drug-specific evidence from oncology-exposome associations and from general ecological mechanisms established in antibiotic, infection, or non-antibiotic-drug models.

### 1.1 Scope, operational definitions, and evidence hierarchy

The term dysbiosis is used here descriptively for a deviation in community composition or diversity. Such a deviation is not automatically harmful. Collateral microbiome injury is a narrower conceptual framework denoting a treatment-associated loss of microbial load, ecological function, spatial organization, colonization resistance, or recovery capacity that is not necessary for tumor killing. Ecological failure refers to the functional consequence of that injury: the community no longer performs one or more protective tasks, such as maintaining anaerobic metabolism, excluding pathobionts, preserving spatial segregation, or recovering before the next perturbation. The concept is intended to sharpen causal and clinical questions; it is not proposed as a currently validated diagnostic entity.

We propose three minimum criteria for an injury claim. First, there should be a documented treatment-associated change in at least one prespecified domain: absolute load, viable burden, ecological function, spatial organization, or recovery capacity. Second, the change should exceed expected within-person temporal variation and should not be fully explained by measured co-exposures. Third, it should have a plausible link to loss of host or ecosystem function, supported where possible by temporality, strain tracking, functional measurements, mediation analysis, or experimental transfer. No universal numerical threshold can yet be justified. Thresholds should be defined prospectively for the measurement platform, treatment setting, and clinical endpoint, preferably relative to each patient’s pretreatment trajectory rather than to a population-average healthy control.

Three evidence levels are used throughout the review. Direct chemotherapy evidence (D) links a defined cytotoxic agent or regimen to a microbial or host-microbiome effect under measured or clinically plausible exposure. Oncology-exposome evidence (O) is obtained during cancer treatment but cannot separate chemotherapy from antibiotics, hospitalization, nutritional change, mucosal injury, or other time-varying exposures. General ecological evidence (G) derives from non-chemotherapy drug screens, antibiotic perturbation, infection models, or basic colonization-resistance studies and is used only as a mechanistic prior. These levels indicate the principal basis of inference, not a formal risk-of-bias score (Table 1).

**TABLE 1:** Operational definitions and evidence levels used in this review.

Term or evidence level	Operational meaning	Preferred measurements and inference boundary
Dysbiosis	A descriptive deviation in composition, diversity, or community structure; it does not by itself establish harm or causality.	Alpha/beta diversity and differential abundance may describe change, but should be interpreted with absolute load, function, and exposure history.
Collateral microbiome injury	Treatment-associated loss of microbial load, function, spatial organization, colonization resistance, or recovery capacity that is not required for antitumor activity.	Prespecify the affected domain; demonstrate change beyond within-person variation and measured co-exposures; link the change to host or ecosystem dysfunction.
Ecological failure	Loss of a protective ecosystem function, such as anaerobic metabolism, pathogen exclusion, spatial segregation, or recovery before the next cycle.	Measure metabolites or flux, viable pathogen burden, source-resolved translocation, and longitudinal recovery; taxonomic change alone is insufficient.
Colonization resistance and domination	Colonization resistance is the community-level exclusion of invading pathogens and resident pathobionts. Domination is a high-burden or high-relative-abundance state that may indicate reduced competition.	Combine relative abundance with total and taxon-specific absolute load. Distinguish expansion of the dominant organism from depletion of competitors.
Oralization/niche coalescence	Breakdown of normal body-site segregation, allowing oral organisms to appear and potentially establish in the distal gut.	Detection in stool is not equivalent to colonization. Require persistence, strain identity, viability, and ideally evidence of functional integration.
Resilience and ecological memory	Resilience is return toward the individual pretreatment state. Ecological memory means that the response to a later cycle depends on prior perturbations and survivors.	Use dense longitudinal sampling, distance-to-baseline, rate of return, load, strain composition, metabolites, and pathogen-exclusion assays. Persistent change is not proof of an alternative stable state.
D: direct chemotherapy evidence	A defined drug or regimen is linked to a microbial or host-microbiome mechanism under measured or clinically plausible exposure.	Supports agent-specific inference; strength depends on pharmacokinetic relevance, community context, temporality, and mechanistic perturbation.
O: oncology-exposome evidence	Microbiome change is observed during cancer treatment with chemotherapy plus antibiotics, hospitalization, dietary change, mucosal injury, or supportive medications.	Supports association with the treatment episode, not chemotherapy-specific causality. Model co-exposures as time-varying variables.
G: general ecological evidence	Mechanisms established in antibiotic, infection, non-antibiotic-drug, or basic ecological models.	Provides a testable mechanistic prior. It should be labeled as extrapolation and validated drug-by-drug and setting-by-setting.

The evidence levels identify the dominant basis of inference and are not formal quality grades. Biomass, absolute bacterial load, and viable microbial load are related but non-interchangeable measurements.

### 1.2 Literature search and evidence selection

This Mini Review used a structured narrative search rather than a systematic-review protocol. PubMed/MEDLINE and ClinicalTrials.gov were searched from database inception through 24 August 2026 using combinations of chemotherapy, cancer, gut microbiome, mucositis, intestinal barrier, colonization resistance, pathobiont, domination, bacteremia, resilience, recovery, fecal microbiota transplantation, probiotic, metagenomics, and metabolomics. Reference lists of recent reviews and key mechanistic papers were also screened. Longitudinal human studies and interventional trials were prioritized for clinical inference; animal, organoid, *ex vivo*, and bacterial-genetic studies were used to assess mechanism. General antibiotic or ecological studies were included only when they defined a specific function that chemotherapy might plausibly remove. Studies limited to pretreatment response prediction without analysis of treatment-associated microbiome change were not central to the synthesis. Because regimens, outcomes, and analytical methods were highly heterogeneous, no pooled effect estimate or formal risk-of-bias score was attempted.

## 2 How chemotherapy perturbs the gut microbiome?

### 2.1 Direct drug–microbe interactions

A direct microbiome effect requires biologically relevant microbial exposure. Oral administration does not guarantee colonic exposure, and intravenous administration does not exclude it. Absorption, biliary or intestinal secretion, enterohepatic recirculation, transit, luminal dilution, mucosal binding, and active-metabolite formation determine the concentration experienced by microbes. Nominal dose and plasma concentration are therefore poor substitutes for measured intestinal exposure. Strong direct evidence should show that a drug or metabolite reaches the relevant compartment at a concentration that changes bacterial viability, transcription, metabolism, or competitive fitness in a community-relevant context.

Irinotecan provides the clearest example of microbiota-mediated local drug reactivation, but not of proven community-wide antibacterial selection. Host-glucuronidated SN-38 is excreted into the intestine as SN-38G, where bacterial β-glucuronidases regenerate active SN-38 and promote epithelial toxicity and delayed diarrhea. Selective enzyme inhibition reduced intestinal injury in mice without broadly killing commensals, preserved antitumor activity, limited a downstream Enterobacteriaceae bloom, and permitted dose intensification (Wallace et al., 2010; Bhatt et al., 2020). A translational study further showed that susceptibility to irinotecan diarrhea could be transferred by fecal microbiota, with expansion of *Bacteroides intestinalis* and production of indole-3-acetate impairing epithelial renewal through PI3K-Akt signaling (Hou et al., 2026). These studies establish microbiota-to-drug and microbiota-to-host causality. They do not, by themselves, demonstrate that irinotecan directly remodels the whole gut community.

Fluoropyrimidines and methotrexate provide more direct evidence of bacterial pharmacological stress, although the evidence level differs by agent. Methotrexate inhibits conserved folate-dependent pathways and produces strain-dependent community effects (Nayak et al., 2021). In colorectal-cancer isolates, 5-fluorouracil inhibited *Fusobacterium nucleatum*, whereas resistant *Escherichia coli* depleted the drug and protected otherwise susceptible bacteria and tumor cells (LaCourse et al., 2022). *Anaerostipes hadrus* can metabolize 5-fluorouracil through bacterial dihydropyrimidine pathways (Liu et al., 2024). In 56 patients receiving capecitabine, longitudinal metagenomics and bacterial genetics linked enrichment of microbial menaquinone-biosynthesis genes to fluoropyrimidine tolerance, with stool pathway and metabolite levels associated particularly with lower peripheral sensory neuropathy (Hillege et al., 2025). These findings support drug-specific ecological filtering and microbial modification of the filter, but they do not justify a class-wide claim for all cytotoxic agents.

Negative community-level evidence is equally informative. In a host-free human colon simulator, 5-fluorouracil and SN-38 caused limited changes in community composition and fermentation at the tested concentrations (Vanlancker et al., 2017). Direct antibacterial activity observed in monoculture may therefore be buffered by degradation, bioaccumulation, cross-protection, spatial refuges, or insufficient luminal exposure. The direct-effect literature supports an agent-by-agent comparative framework: irinotecan is strongest for microbial drug reactivation, methotrexate and 5-fluorouracil for conserved bacterial targets and strain-specific metabolism, capecitabine for longitudinal functional selection, and many other agents for plausibility rather than demonstrated gut-community injury.

### 2.2 Host-mediated habitat remodeling

Chemotherapy can reshape the microbiome even when luminal drug concentrations are too low for direct inhibition. The epithelium, mucus layer, immune system, diet, and motility constitute ecological filters that determine which organisms can persist. Cytotoxic injury to proliferating crypt cells reduces epithelial renewal, disrupts tight junctions, changes antimicrobial-peptide release, and exposes host-derived nutrients. Mucus depletion alters attachment sites and spatial separation. Reduced enteral intake removes fermentable substrates, while diarrhea, constipation, opioids, antiemetics, laxatives, and parenteral nutrition alter transit and washout.

The redox component of this model is supported mainly by general ecological evidence. Depletion of butyrate-producing anaerobes can reduce epithelial PPAR-γ signaling and increase epithelial oxygenation, while inflammation supplies nitrate and other electron acceptors that favor facultative Enterobacteriaceae (Winter et al., 2013; Byndloss et al., 2017). This mechanism was not originally established in chemotherapy-treated patients and should be treated as a testable prior rather than a proven universal pathway. Chemotherapy-specific models, however, show that host injury can generate microbial signals. Purines released from apoptotic epithelial cells reprogrammed Enterobacteriaceae respiration and delayed tissue recovery (Anderson et al., 2024). In oxaliplatin models, an IFN-γ-IDO1-kynurenine program depleted *Lactobacillus johnsonii* and amplified TNF-α/JNK-dependent epithelial apoptosis (Xie et al., 2026). Transfer of microbiota from oxaliplatin-exposed mice worsened toxicity in recipients through reduced macrophage-derived interleukin-10 (He et al., 2024). These studies support a self-reinforcing host-microbiome circuit, while leaving the initial balance between direct drug exposure and habitat injury agent-specific.

Human studies most often provide oncology-exposome evidence. In a small cohort designed to minimize concurrent antibacterial exposure, myeloablative chemotherapy was associated with diversity loss and depletion of health-associated taxa, showing that change is not entirely reducible to antibiotics (Montassier et al., 2015). In acute myeloid leukemia, however, carbapenem exposure tracked strongly with oral and stool diversity loss, and cumulative antibiotic days predicted temporal instability (Galloway-Peña et al., 2016, 2017). Intensive antileukemia chemotherapy and allogeneic transplantation produce partly similar low-diversity, *Enterococcus*-dominated states, but their treatment histories and immune contexts differ (Rashidi et al., 2020a). These data support a composite treatment-episode model rather than drug-specific attribution.

This distinction also limits generalization across cancer types. Intensive leukemia regimens combine prolonged hospitalization, profound neutropenia, repeated broad-spectrum antibiotics, and marked dietary disruption. Conventional solid-tumor chemotherapy may involve outpatient treatment, shorter antibiotic exposure, and different levels of epithelial injury. Solid-tumor studies of capecitabine, irinotecan, oxaliplatin, and postoperative gastric-cancer chemotherapy provide important agent- and cycle-specific observations, but they do not reproduce the full leukemia exposome. The causal contribution of chemotherapy should therefore be estimated within each regimen and care setting.

## 3 From compositional change to ecological failure

Section 3 follows a defined progression. Loss of commensal functions reduces the mechanisms that exclude pathogens; weakened colonization resistance permits expansion of resident pathobionts or establishment of ectopic organisms; domination and barrier failure then create conditions in which translocation becomes possible. Resistome or virulence enrichment is considered a conditional downstream consequence of this ecological shift, not an independent proof of chemotherapy-induced injury.

### 3.1 Loss of commensal function

A reduction in Shannon diversity can arise from depletion of rare taxa, domination by one organism, reduced total bacterial load, or altered evenness, each with different implications. Functional assessment should therefore focus on fermentation of dietary substrates, short-chain fatty acid production, primary-to-secondary bile-acid conversion, occupation of spatial niches, nutrient competition, cross-feeding, maintenance of low redox potential, stimulation of mucus and antimicrobial defenses, and resistance to invasion.

Obligate anaerobes contribute to several of these functions. Butyrate supports colonocyte metabolism and barrier integrity, fermentation products can acidify the lumen, and secondary bile acids contribute to resistance against *Clostridioides difficile* (Buffie et al., 2015; Theriot et al., 2016). Dense interaction networks also provide functional redundancy. Repeated chemotherapy and antibiotics may erode this redundancy before a large taxonomic shift becomes obvious, whereas a compositionally altered community may remain clinically stable if critical functions are preserved.

Multi-omics studies illustrate why pathway-level measurements may be more informative than genus-level lists. During intensive acute myeloid leukemia therapy, durable taxonomic change was accompanied by reduced fecal bacterial amino-acid metabolites and associations among disappearing organisms, barrier dysfunction, and weight loss (Pötgens et al., 2023). During capecitabine treatment, a prominent longitudinal signal was enrichment of menaquinone-biosynthesis genes rather than a universal taxon (Hillege et al., 2025). Gene abundance alone does not establish expression, metabolic flux, or delivery to the host; metatranscriptomics, targeted metabolomics, activity assays, and, where feasible, isotope tracing are needed to demonstrate functional loss or reprogramming.

### 3.2 Loss of colonization resistance

Colonization resistance is the community-level ability to prevent establishment or expansion of exogenous pathogens and resident pathobionts. It emerges from overlapping mechanisms rather than a single protective taxon. Commensals compete for carbon, nitrogen, micronutrients, and attachment sites; produce inhibitory metabolites; transform bile acids; acidify the lumen; stimulate epithelial defenses; and restrict pathogen growth through interaction networks.

Most mechanistic examples were established outside chemotherapy and are therefore general ecological evidence. These include microbiota-mediated intracellular acidification of Enterobacteriaceae, propionate-mediated restriction of *Salmonella*, inflammation-dependent nitrate respiration by *Escherichia coli*, and secondary-bile-acid-mediated resistance to *Clostridioides difficile* (Sorbara et al., 2019; Jacobson et al., 2018; Winter et al., 2013; Buffie et al., 2015). Non-antibiotic drug screens further show that human-targeted medications can preferentially inhibit commensals and open metabolic niches for enteropathogens (Grießhammer et al., 2025; Kumar et al., 2025). Their relevance to cytotoxic treatment must be tested drug-by-drug, but they define functions that chemotherapy or the oncology exposome could plausibly remove.

Domination is more interpretable than a small relative-abundance difference, but it is not equivalent to increased pathogen burden. A taxon may appear dominant because competitors declined while its absolute abundance remained stable. Conversely, absolute pathogen load may increase without a striking proportional change. Quantitative profiling can reverse conclusions based on proportions alone (Vandeputte et al., 2017). Studies should therefore distinguish microbial biomass, absolute bacterial load, taxon-specific load, and viable load, using combinations of spike-in standards, flow cytometry, digital PCR, quantitative culture, or viability-informed assays.

### 3.3 Pathobiont domination, oralization, and niche coalescence

Longitudinal leukemia cohorts repeatedly identify transitions toward *Enterococcus*-, Streptococcaceae-, or Proteobacteria-rich states. In 199 children with acute lymphoblastic leukemia, baseline Proteobacteria predicted febrile neutropenia, while subsequent Enterococcaceae domination predicted fever and diarrhea (Hakim et al., 2018). During acute myeloid leukemia induction, pathogenic-genus domination increased, but carbapenem exposure was a major driver (Galloway-Peña et al., 2016). Specific community changes have also preceded neutropenic fever or bloodstream infection (Rashidi et al., 2020b). These temporal associations are stronger than cross-sectional correlations but remain oncology-exposome evidence unless the responsible exposure and source organism are resolved.

Strain-resolved linkage provides a stronger bridge from intestinal ecology to infection. In transplantation and other high-risk settings, domination can precede bacteremia, and bloodstream pathogens can be identified in the preceding gut microbiome (Ubeda et al., 2010; Taur et al., 2012; Tamburini et al., 2018). During chemotherapy, paired longitudinal stool, oral, and blood sampling with isolate or metagenome-resolved genomes is needed to distinguish endogenous translocation from independent acquisition and to identify whether selection involved a pre-existing gut strain, a hospital-acquired strain, or an oral strain establishing in the distal gut.

Oralization or niche coalescence adds a spatial dimension to injury. In 39 patients undergoing intensive chemotherapy, strain-level analysis of 283 paired oral and fecal samples showed increasing oral-gut overlap over time and greater overlap in patients with intestinal mucositis (Raychaudhuri et al., 2025). A separate longitudinal cohort of 29 patients receiving postoperative chemotherapy for advanced gastric cancer reported increases in gut *Escherichia-Shigella* and *Streptococcus* and reduced oral-gut network complexity, but marker-gene sequencing could not establish strain migration (Xu et al., 2026). Detection of an oral taxon in stool is not equivalent to stable distal colonization. Strong evidence requires persistence, strain identity, viability, and preferably evidence of functional integration. Oralization may be a driver of altered metabolism and inflammation, or it may be a marker of severe habitat disruption; current data support both possibilities.

### 3.4 Conditional resistome and virulence consequences

Pathobiont expansion can enrich resistance and virulence genes simply by increasing the biomass of organisms that carry them. Cytotoxic agents might also select bacterial stress responses, efflux, nucleotide salvage, or DNA-repair functions, but independent chemotherapy-driven selection of the intestinal resistome remains insufficiently demonstrated. In oncology cohorts, broad-spectrum antibiotics are a dominant competing exposure. Among hematopoietic-transplant recipients, broad-spectrum β-lactams were strongly associated with disruption and resistome changes, illustrating the need for time-resolved antibiotic data (Nørgaard et al., 2023). Baseline microbiome and resistome features have also been associated with subsequent resistant colonization or infection in acute myeloid leukemia, but this does not establish chemotherapy as the selecting agent (Batool et al., 2025).

Accordingly, claims that chemotherapy drives antimicrobial resistance should require exposure-resolved metagenomics or isolate genomics, absolute abundance, and localization of a resistance determinant within a clinically relevant strain. The more defensible research question is whether chemotherapy-induced habitat change and antibiotic selection interact additively or synergistically to favor high-risk pathobionts.

## 4 Clinical consequences of chemotherapy-associated ecological injury

### 4.1 Mucositis, diarrhea, and neutropenic enterocolitis

The relationship between microbial change and mucosal injury is bidirectional. Chemotherapy initiates crypt-cell damage and mucositis; the altered habitat then selects a different community that may amplify inflammation, barrier permeability, and metabolic deprivation. Depletion of butyrate producers can reduce trophic support for colonocytes, while pathobiont expansion increases the microbial stimulus presented to a compromised barrier (Byndloss et al., 2017; Hueso et al., 2020). This feedback may prolong injury beyond peak drug exposure, although its relative contribution varies by regimen.

Translational evidence is strongest when host and microbial measurements are collected together. In acute myeloid leukemia patients and a chemotherapy model, induction therapy reduced citrulline, fecal bacterial load, short-chain fatty acids, and diversity, while *Escherichia coli* and *Enterococcus* became dominant; experimental mucus strengthening accelerated epithelial recovery and reduced translocation (Hueso et al., 2020). A prospective study of 65 patients undergoing acute myeloid leukemia induction found lower diversity, increased *Enterococcus faecalis*, depletion of butyrate-producing genera, reduced fecal butyrate, and enterocyte injury measured by plasma citrulline. Neutropenic enterocolitis was accompanied by a local inflammatory program involving JAK-STAT, interleukin-6, and oncostatin-M signaling (Kapandji et al., 2025). These data support an integrated barrier-microbiota model but remain difficult to separate from antibiotics and other intensive-care exposures.

Diarrhea is not a single microbiome phenotype. Irinotecan has a defined bacterial-enzyme mechanism, whereas fluoropyrimidine-, platinum-, and combination-regimen diarrhea may reflect epithelial toxicity, infection, altered bile acids, motility, supportive medications, or community change. Studies should integrate graded mucositis, stool frequency and water content, pathogen testing, drug exposure, barrier markers, and microbial function rather than pool all diarrhea events.

### 4.2 Febrile neutropenia and bloodstream infection

A simplified sequence of chemotherapy-induced dysbiosis followed directly by bacteremia is not supported. A more realistic model is: cytotoxic treatment plus antibiotics, mucosal injury, immune suppression, hospitalization, and nutritional disturbance produce microbiome and barrier alterations; these changes may weaken colonization resistance and permit domination or translocation; infection occurs only when source, barrier, and host-containment conditions align. Central lines, skin, lungs, and oral mucosa remain alternative sources, and empirical antibiotics may alter stool samples before the source is established.

The evidence hierarchy should therefore be explicit. A cross-sectional association between a taxon and infection is weak. A pre-event rise in absolute pathogen load is stronger. Persistent strain-resolved carriage followed by a genetically matched bloodstream isolate is stronger still. Experimental reproduction of translocation after transfer of a treatment-conditioned community provides mechanistic support. Pediatric acute lymphoblastic leukemia and acute myeloid leukemia cohorts show temporality between domination or instability and infectious outcomes, but few chemotherapy studies combine absolute load, barrier measurements, and strain-resolved blood-source matching (Hakim et al., 2018; Galloway-Peña et al., 2016, 2017; Rashidi et al., 2020b). The plausibility of colonization-resistance failure therefore exceeds the certainty of attribution to a specific chemotherapeutic agent.

### 4.3 Treatment tolerance and continuity

Collateral microbiome injury may affect cancer care without microbiologically documented infection. Persistent diarrhea, mucositis, anorexia, weight loss, and systemic inflammation can cause dose reduction, treatment delay, prolonged hospitalization, or transition to a less intensive regimen. In an acute myeloid leukemia multi-omics cohort, enduring microbial and metabolite changes were associated with barrier dysfunction and body-weight loss (Pötgens et al., 2023). During capecitabine treatment, baseline and treatment-responsive microbial pathways were associated with adverse-event profiles and dosing changes (Hillege et al., 2025). These studies identify clinically relevant ecosystem markers but do not prove that microbiome modification would prevent toxicity.

The marker-mediator distinction is therefore critical. A low-diversity community may record accumulated treatment intensity, whereas transfer experiments showing that a treatment-conditioned microbiota worsens toxicity demonstrate mediation in specific models (He et al., 2024). Future human studies should prespecify mediation analyses and pair them with mechanistic validation.

### 4.4 Safeguarding antitumor efficacy

Microbial metabolism and systemic immunity can influence chemotherapy efficacy, as shown by cyclophosphamide-, platinum-, and gemcitabine-related studies (Viaud et al., 2013; Iida et al., 2013; Geller et al., 2017). These studies address microbiota effects on therapy rather than chemotherapy-induced gut injury and are therefore secondary to the present review. Their main implication is a safety constraint: any microbiome-sparing intervention must reduce intestinal or ecological injury without decreasing tumor control or inadvertently protecting tumor-associated bacteria. Selective bacterial β-glucuronidase inhibition is instructive because it reduced irinotecan toxicity without compromising antitumor activity in mice (Bhatt et al., 2020).

## 5 Resilience, ecological memory, and persistent altered states

A single pretreatment/post-treatment comparison cannot characterize recovery. Resistance is minimal displacement during treatment. Resilience is return toward the individual pretreatment state after displacement. Incomplete recovery is partial return with persistent taxonomic, strain-level, functional, or spatial differences. Ecological memory means that a later perturbation acts on a community altered by previous cycles. An alternative stable state is a stronger theoretical claim requiring evidence that a new configuration is maintained by feedbacks and persists after the perturbation is removed (Dethlefsen and Relman, 2011; Lozupone et al., 2012; Costello et al., 2012; de la Cuesta-Zuluaga et al., 2024). Current chemotherapy data are more appropriately described as persistent altered states or patterns consistent with ecological memory.

Chemotherapy is a sequence of perturbations rather than a single pulse. The first cycle can remove susceptible organisms and reduce redundancy; the next cycle therefore begins from a different ecological baseline. In acute leukemia, re-induction or salvage therapy was associated with larger departures from baseline and *Enterococcus* expansion, a pattern consistent with ecological memory but not proof of a stable-state transition (Rashidi et al., 2019). In 52 acute myeloid leukemia patients with 410 longitudinal samples, post-discharge communities remained highly dissimilar to baseline after major antibiotic pressure had declined (Rashidi et al., 2022). This demonstrates persistent reorganization after the composite treatment episode, not chemotherapy-specific causality.

Alpha diversity remains a useful descriptive component of recovery, but it is insufficient alone. Diversity can increase through replacement by new strains, expansion of low-abundance organisms, or oral-to-gut colonization, while metabolic function or pathogen exclusion remains impaired. Conversely, composition may remain different while function is restored through redundancy. Recovery should therefore be operationalized along at least five axes: total and taxon-specific absolute load; viable microbial burden where clinically relevant; taxonomic and strain similarity to the individual baseline; metabolic and ecological function; spatial segregation and resistance to pathobiont expansion or invasion.

Sampling time is equally important. Discharge may capture the nadir or early succession rather than a stable endpoint. Dense sampling during treatment should be followed for months and should include the interval before the next chemotherapy, transplantation, or immunotherapy exposure. Recovery can then be modeled by distance-to-baseline, rate of return, variance over time, and the proportion of prespecified functions restored. These measures provide operational definitions that can be compared across regimens without assuming that return to a population-average healthy composition is necessary.

## 6 Causal attribution and translational priorities

### 6.1 Study design and multidimensional measurement

The phrase chemotherapy-induced dysbiosis implies a single exposure, but most oncology cohorts contain time-varying antibacterial and antifungal therapy, proton-pump inhibitors, glucocorticoids, opioids, antiemetics, laxatives, enteral or parenteral nutrition, hospitalization, infection, dietary restriction, prior treatment, surgery, and radiotherapy. Host variables are major confounders in human microbiome studies generally and are amplified in intensive oncology (Vujkovic-Cvijin et al., 2020).

Several design principles can improve attribution. Drug and supportive-care exposure should be recorded daily with dose, route, timing, and spectrum; anti-anaerobic antibiotic days should be analyzed separately. Sampling should be anchored to biologically meaningful phases: before therapy, after drug exposure but before empirical antibiotics when feasible, at mucosal and neutrophil nadirs, during recovery, between cycles, and in long-term follow-up. Regimens should not be pooled simply because they are all chemotherapy. Capecitabine, irinotecan, high-dose methotrexate, cytarabine-anthracycline induction, and conditioning regimens generate different intestinal exposures and host injuries.

Relative-abundance data are compositional and should be paired with absolute measurements (Gloor et al., 2017). Microbial biomass refers broadly to total microbial material; absolute bacterial load estimates bacterial cells or gene copies per unit sample; viable load estimates living organisms capable of growth or metabolic activity. These quantities are related but not interchangeable. Stool water content, sample mass, spike-in recovery, and pre-analytical handling should be recorded. Shotgun metagenomics should be strain-resolved when source tracking or acquisition is the question, and paired oral, stool, and bloodstream isolates should be compared whenever translocation is proposed.

No biomarker is currently validated specifically for collateral microbiome injury. Plasma citrulline is a useful marker of enterocyte mass and mucosal injury but is not microbiome-specific. Fecal short-chain fatty acids, secondary bile acids, absolute obligate-anaerobe load, pathogen burden, oral-gut strain sharing, and distance-to-baseline are candidate components of a composite panel rather than stand-alone diagnostic tests. Human studies should integrate these measures with drug concentrations, diet, transit, permeability, inflammation, and prespecified clinical outcomes. Reporting should follow microbiome standards such as STORMS, including negative results, batch effects, contamination controls, and covariate definitions (Mirzayi et al., 2021).

### 6.2 Interventions stratified by current evidence

No microbiome-restoration intervention is established as standard supportive care across chemotherapy settings. Clinically tested approaches should therefore be distinguished from mechanistically promising and hypothesis-generating strategies. In a randomized, double-blind trial of 104 patients receiving chemotherapy for advanced gastric or esophageal cancer, *Bifidobacterium animalis* subsp. lactis V9 improved several quality-of-life domains and produced targeted taxonomic and metabolic shifts without changing global alpha or beta diversity (Yang et al., 2026). This supports a function-focused intervention in a defined solid-tumor setting, but it does not establish broad probiotic efficacy across regimens.

In allogeneic transplantation and acute myeloid leukemia, a randomized phase II trial of encapsulated third-party fecal microbiota transplantation improved post-antibiotic diversity recovery, restored depleted obligate anaerobes, and reduced expanded genera, but did not significantly reduce infections (Rashidi et al., 2023). The phase 1b CIMON study of the pooled oral microbiotherapeutic MaaT033 in 21 acute myeloid leukemia patients after intensive chemotherapy and antibiotics reported acceptable safety, donor-community engraftment, and microbiota reconstruction; confirmatory controlled trials are needed (Malard et al., 2025). A phase 1 study is also evaluating oral fecal microbiota transplantation with gemcitabine and nab-paclitaxel in advanced pancreatic ductal adenocarcinoma (ClinicalTrials.gov NCT06393400). These interventions are clinically tested or under investigation, not established care.

Mechanistically promising strategies include targeted inhibition of bacterial drug-reactivating enzymes, protection of mucus and epithelium, maintenance of enteral substrates, and defined metabolite replacement. Their value depends on matching the intervention to the missing function and demonstrating preserved antitumor efficacy. Hypothesis-generating approaches include broad fecal microbiota transplantation, live biotherapeutic consortia, and strain-specific decolonization during profound neutropenia. In this setting, microbial translocation, donor screening, antimicrobial-resistance transfer, and strain traceability are central safety constraints. Intervention timing may be as important as composition: after mucosal and neutrophil recovery but before the next treatment cycle may provide a more favorable window than the nadir.

A successful intervention should meet a dual endpoint: restoration of a prespecified ecological function or clinically relevant outcome without increasing infection risk or reducing antitumor activity. This standard is more stringent than producing an increase in alpha diversity.

## 7 Conclusion

Chemotherapy-associated microbiome change should not be reduced to a list of taxa that rise or fall. Direct drug-microbe interactions are supported for selected agents, whereas much of the human longitudinal literature reflects the broader oncology exposome. General ecological mechanisms provide valuable hypotheses but must be labeled as extrapolations and tested within specific regimens. When treatment-associated pressures remove core anaerobic functions, weaken colonization resistance, erode spatial segregation, or impair recovery, compositional dysbiosis may progress to ecological failure. The clinically relevant outcomes are not diversity loss itself, but loss of pathogen exclusion, amplification of mucosal injury, possible translocation, impaired treatment tolerance, and failure to restore a functionally protective ecosystem before the next cycle. Progress will require exposure-resolved longitudinal designs, absolute and viable-load measurements, strain tracking, functional assays, host-barrier markers, and evidence-stratified interventions. The practical aim is not to preserve a fixed taxonomic baseline at all costs, but to protect or rebuild the ecological functions that allow effective cancer therapy to be delivered safely (Figure 1).

**FIGURE 1:**
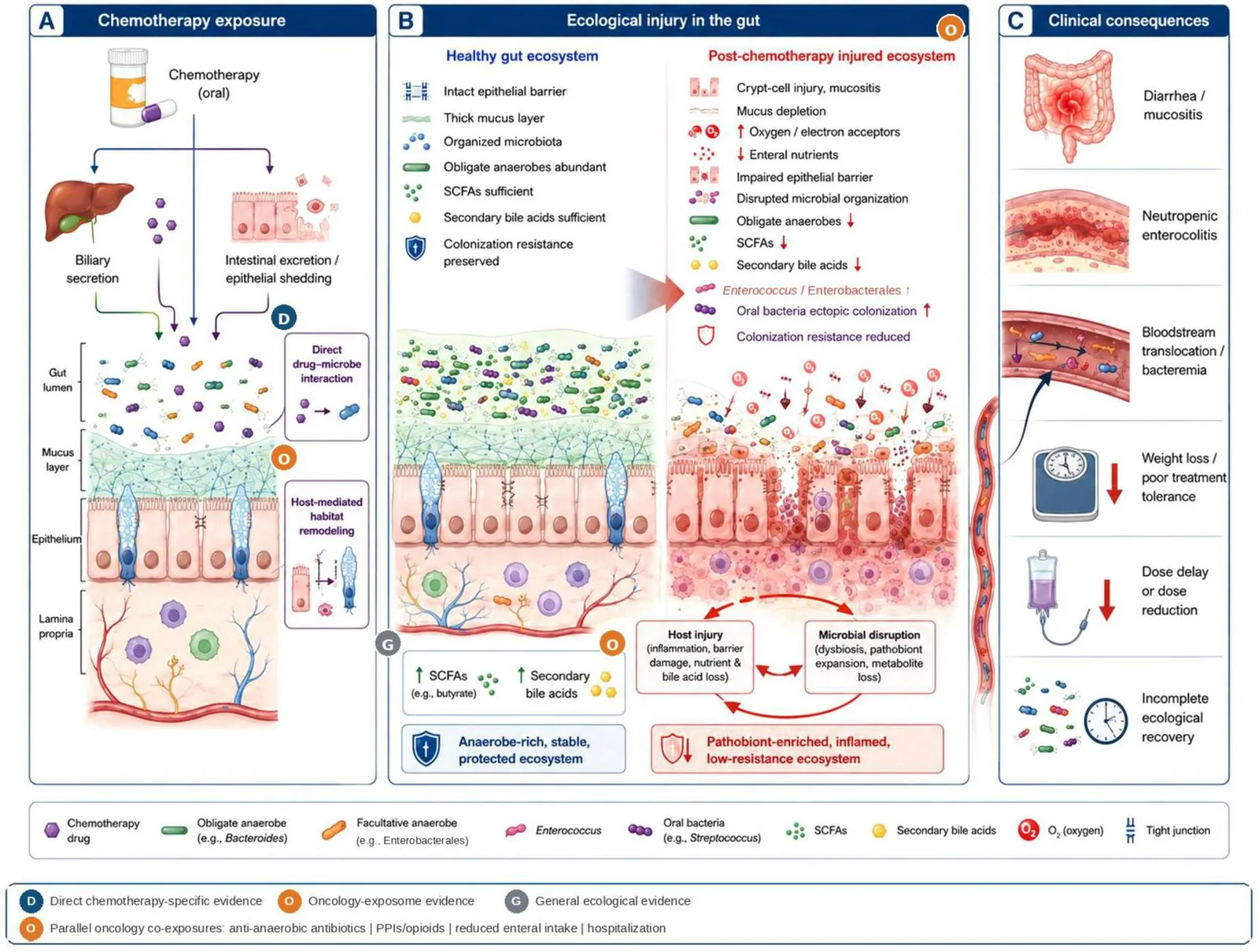
Mechanistic model of collateral microbiome injury during cancer chemotherapy. **(A)** Chemotherapeutic agents may reach the intestinal ecosystem after oral administration, biliary secretion, intestinal excretion, epithelial shedding, or enterohepatic recirculation. Microbiome perturbation may arise from direct drug-microbe interactions or from host-mediated habitat remodeling. **(B)** In the healthy gut, an intact barrier, thick mucus, obligate anaerobes, short-chain fatty acids, secondary bile acids, and nutrient and spatial competition support colonization resistance. Chemotherapy-associated crypt injury, mucositis, mucus depletion, reduced enteral substrate, and altered redox conditions may deplete obligate anaerobe functions and favor *Enterococcus*, Enterobacterales, and ectopic oral organisms. Host injury and microbial disruption may then reinforce one another. **(C)** The resulting ecological failure may contribute to diarrhea and mucositis, neutropenic enterocolitis, possible microbial translocation and bloodstream infection, weight loss, poor treatment tolerance, dose delay or reduction, and incomplete recovery. Evidence labels indicate the principal basis of inference: D, direct chemotherapy-specific evidence; O, oncology-exposome evidence in which chemotherapy co-occurs with antibiotics, hospitalization, nutritional disturbance, or supportive medications; and G, general ecological evidence extrapolated from other perturbation models. These labels are not formal quality grades, and pathways are not equally established in chemotherapy-treated patients. Parallel oncology co-exposures are shown explicitly because they may be dominant drivers of the observed microbiome state. Blue and green indicate homeostatic or protective features; orange and red indicate injury, inflammation, or pathobiont-associated processes. Arrows indicate proposed directions of interaction, and bidirectional arrows indicate reciprocal reinforcement. SCFAs, short-chain fatty acids; PPIs, proton-pump inhibitors.
